# Serum Calprotectin in the Evaluation of Gastrointestinal Diseases: An Ace up Your Sleeve?

**DOI:** 10.3390/medicina60050762

**Published:** 2024-05-05

**Authors:** Angela Saviano, Alessio Migneco, Mattia Brigida, Carmine Petruzziello, Christian Zanza, Gabriele Savioli, Francesco Franceschi, Veronica Ojetti

**Affiliations:** 1 Emergency Department, Policlinico Universitario A. Gemelli IRCCS, 00168 Rome, Italy; angela.saviano@unicatt.it (A.S.); alessio.migneco@policlinicogemelli.it (A.M.); francesco.franceschi@unicatt.it (F.F.); 2 Department of Medicine, Catholic University of the Sacred Heart, 00168 Rome, Italy; 3 Gastroenterology Unit, Policlinico Universitario Tor Vergata, 00133 Rome, Italy; 4 Department of Emergency Medicine, Ospedale San Carlo di Nancy, GVM Care and Research, 00165 Rome, Italy; carminepetruzziello@live.it; 5 Geriatric Medicine Residency Program, University of Rome “Tor Vergata”, 00133 Rome, Italy; christian.zanza@live.it; 6 Emergency Medicine and Surgery, IRCCS Fondazione Policlinico San Matteo, 27100 Pavia, Italy; gabrielesavioli@gmail.com

**Keywords:** serum calprotectin, fecal calprotectin, microbiota, IBD, autoimmune diseases

## Abstract

*Background*: Calprotectin (CP) is a calcium- and zinc-binding protein that plays a key role in innate immunity and in the recruitment of inflammatory cells. CP can be detected both in serum and in fecal samples. Serum CP (sCP) is more specific for autoimmune diseases, while fecal CP (fCP) has been well investigated for gastrointestinal diseases. Few studies have shown the clinical effectiveness of sCP as an acute-phase biomarker for gastrointestinal diseases. *Aim*: The aim of this narrative review is to discuss the role of sCP as a useful alternative biomarker of the acute-phase activity of gastrointestinal diseases and as a possible tool for screening and monitoring these diseases. *Material and Methods*: We searched original articles, abstracts, reviews, case reports, and clinical trials on PubMed^®^, Up-to-Date^®^, and Medscape^®^ in the last ten years. *Conclusion*: We found that sCP could represent a useful biomarker in the evaluation of the inflammatory stage in patients with immune-mediated gastrointestinal diseases, but more studies are needed to promote its routine use in clinical practice as a diagnostic and prognostic biomarker as a replacement for fCP.

## 1. Introduction

Calprotectin (CP) is a heterodimeric complex composed of two S100 proteins, first isolated from the bovine brain in 1965 [1]. It can be detected in fecal and serum samples [2]. Fecal detection is commonly used as a specific and sensitive marker for the evaluation of inflammatory bowel diseases (IBDs) (for both diagnosis and follow-up) [3]. Few studies have investigated the role of serum calprotectin (sCP) as a clinical biomarker for the diagnosis, disease relapse, and response to treatment of gastrointestinal diseases [3]. Despite the fact that gastrointestinal diseases such as IBD greatly influence patients’ lives, there is an urgent demand for non-invasive and accurate blood biomarkers to guide treatment and estimate their recurrence and prognosis [4]. C-reactive protein (CRP) and the erythrocyte sedimentation rate (ESR) are extensively used serum indicators of the “acute phase”. They are sensitive and specific for gut inflammation [5]. CRP and ESR levels are frequently elevated in other conditions, such as infections and autoimmune disorders [5]. sCP has been investigated as a biomarker of the acute phase in patients with IBD exacerbations [3]. A significant association has been found between sCP and CRP levels in these patients [5]. The incidence of IBD has grown, along with its financial and health impacts. Colonoscopy and histological examination are the gold standard for its diagnosis. Colonoscopy is an invasive, expensive, and time-consuming procedure for patients, hence the need to search for easy, cheap, and non-invasive biomarkers to follow up on patients, monitor disease activity, and predict outcomes. Some studies have been conducted on the pediatric population, too [6]. Children and adolescents are often noncompliant in collecting fecal samples, with suboptimal monitoring. Malham et al. [7] analyzed the data of 30 pediatric patients with suspected or confirmed ulcerative colitis (UC), concluding that sCP can be a useful biomarker of disease activity, especially in cases where fCP measurements are difficult to obtain due to patients’ compliance.

### Calprotectin: From Biology to Clinical Practice Use

Calprotectin (CP) is a calcium- and zinc-binding protein composed of two small proteins, named S100A8 and S100A9 [8]. It is produced or released by immune cells as activated neutrophils and monocytes but also endothelial cells. The studies in the literature show that its levels increase rapidly in the presence of microorganisms such as bacteria. Its expression is activated by pathogen-associated molecular patterns (PAMPs) and damage-associated molecular patterns (DAMPs) [9]. CP plays a crucial role in the recruitment of inflammatory cells. It contains direct bactericidal properties mediated by the chelation of ions such as Mn^2+^ and Zn^2+^ [10]. Furthermore, CP acts as a ligand for Toll-like receptor 4 (TLR-4), which transmits the danger-damages signals. Moreover, CP triggers phagocyte nicotinamide adenine dinucleotide phosphate (NADPH) oxidase activation and the inflammatory pathways mediated by tumor necrosis factor α (TNF-α) involved in lipopolysaccharide toxicity. It promotes the rearrangement of the tubulin cytoskeleton that is necessary for the migration of activated neutrophils and monocytes [11]. The data in the literature reveal that CP is involved in a variety of biological processes, such as cell homeostasis, the transduction of signals, and inflammation. Moreover, it activates signaling pathways such as the mitogen-activated protein kinase (MAPK) pathway, the NADPH oxidase phagocytes system, and nuclear factor-κB (NF-kB) [12]; it upregulates neutrophil activities after being stimulated by lipopolysaccharides or chemokines in response to pathogens. CP weakens the cell–cell contacts and promotes leukocyte extravasation [12]. In the presence of excess Ca^2+^, the heterodimers S100A8 and S100A9 aggregate to heterotetramers, which are more resistant to proteolysis. To summarize, the functions of CP can be distinguished as intracellular and extracellular. Intracellular functions are mainly mediated by the binding with TLR4 and receptor for advanced glycation products (RAGE) and NF-kB, triggering the amplification of proinflammatory cytokines [13]. The presence of calcium facilitates microtubule aggregation and the activation of the NADPH oxidase pathway, with the production of reactive oxygen species (ROS). Extracellular functions consist of the activation of leukocytes, the promotion of chemotaxis, and the activation of endothelial cells. Moreover, CP leads to the adhesion of neutrophils to vascular endothelial cells, with antimicrobial action but a risk of clot formation. CP can be assessed in serum samples (sCP) or fecal samples (fCP). The serum levels of CP (sCP) are usually estimated to be below 1 ng/mL in healthy subjects, while fecal levels (fCP) are below 50 µg/gr [14]. sCP is more specific in autoimmune diseases compared to fCP, and showed a high sensitivity and specificity in discriminating between active forms of inflammatory bowel disease (IBD) and irritable bowel syndrome (IBS) [4].

## 2. Materials and Methods

This narrative review includes studies published in the last 10 years (no language restrictions were applied). The main research topics involved the correlation between sCP and autoimmune diseases, with a focus on gastrointestinal ones. We searched for clinical trials, reviews, observational studies, and case reports. We extracted data in view of the abstract, the period of research, the type of study, and the title. We searched on PubMed^®^, Web of Science^®^, UptoDate^®^, and Cochrane^®^. No ethical approval was needed. We searched for the following principal words: calprotectin AND autoimmune diseases, sCP AND gastrointestinal diseases AND/OR IBD; fCP and gut microbiota AND immune system, sCP AND fCP AND/OR autoantibodies, sCP AND/OR gut barrier, dysbiosis AND sCP AND fCP. 

## 3. Results

### 3.1. Serum Calprotectin (sCP) and Autoimmune Diseases

**Figure 1:**
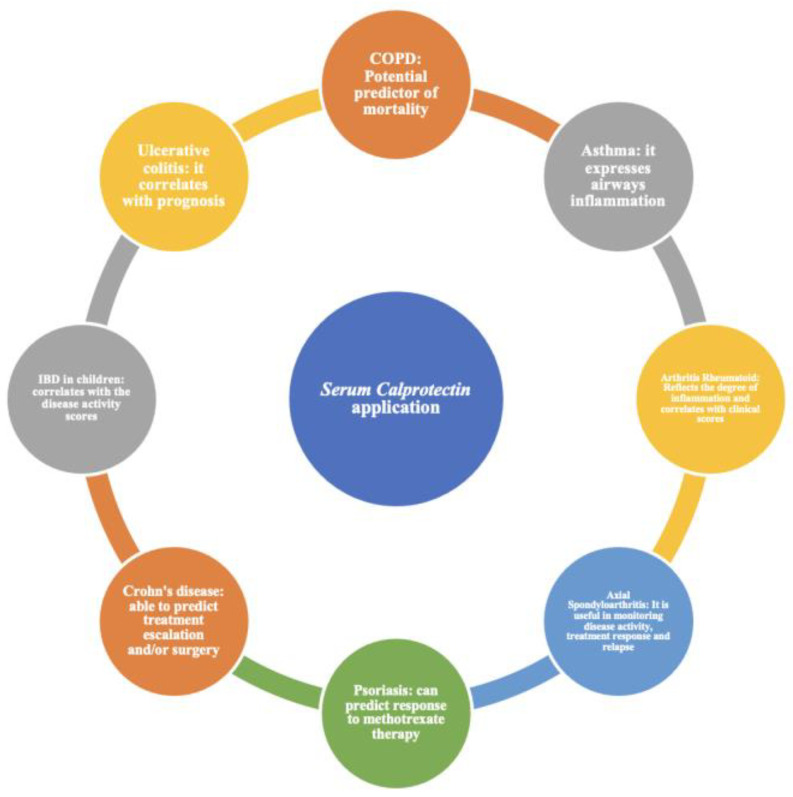
Serum calprotectin (sCP) utility and application in immune-mediated diseases [4,39,40,41,42,43].

sCP has been studied as an acute-phase protein in autoimmune diseases [15] (Figure 1) such as rheumatoid arthritis (RA), myasthenia gravis (MG), Hashimoto’s disease, autoimmune vasculitis, lupus erythematous syndrome (SLE), Sjogren’s syndrome, myasthenia gravis (MG), and hemolytic anemia [16,17,18,19,20]. sCP plays an important role in the regulation of adaptive immune responses. In particular, studies in the literature show that sCP promotes the induction of CD8-T cells that have pre-programmed cytotoxic functions. Further, sCP regulates the overexpression of dendritic cells, acts as an endogenous ligand of CD69, and is a costimulatory raiser of CD40/CD40L, increasing the activation of T-cells [8]. sCP directly bound white cells such as macrophages, granulocytes, and monocytes and promoted the production of pro-inflammatory cytokines such as TNF-α, IL-1, and IL-6, correlating with inflammation [16,17]. Further, sCP levels were positively associated with both CRP values and radiological progression in patients with RA with a potential prognostic value [16,20]. sCP is not widely used compared with fCP. Its blood dosage can be affected by variables such as type of anticoagulant, storage time, and temperature, with different results. Some studies that correlate sCP and autoantibodies in autoimmune diseases showed that high levels of sCP have been associated with positive autoantibodies (Ab-anti-dsDNA, anti-SSA, anti-Ro60, AchR-Ab, Musk-Ab, LRP4-Ab, levels of MPO and PR3…). Some studies support the association between sCP and disease activity in both RA and axial spondyloarthritis (axSpA) [21]. In addition, there is a positive association between sCP and traditional acute-phase proteins and ultrasound and radiographic imaging of the progression of joint inflammation in patients affected by RA [22]. Many mechanisms may contribute to joint damage, such as activation of the TLR-4 signaling or NF-kB and p38 MPAK pathways mediated by sCP, which leads to the secretion of pro-inflammatory cytokines [16,20,21]. Other research shows that sCP correlates with treatment response in patients with RA [23]. Furthermore, high sCP levels predict future erosive joint damage [22]. In patients with SLE arthritis, sCP levels were higher than in patients without arthritis [24]. sCP has been considered a predictive biomarker of both local and systemic inflammation in chronic inflammatory rheumatic diseases [18]. Interestingly, high levels of sCP have been found in Crohn’s disease (CD) and UC, associated with positive anti-Saccharomyces cerevisiae antibodies (ASCA) and perinuclear ANCA (pANCA) [25,26].

### 3.2. Serum Calprotectin (sCP) and Inflammatory Bowel Diseases 

Inflammatory bowel diseases (IBDs) are chronic inflammatory disorders that include CD and UC [27]. Their incidence is increasing in industrialized countries, and they can affect people of any age. Some factors, such as genetic predisposition, early antibiotic exposure, gut microbiota, environmental factors, and the innate and adaptive immune system could lead to the onset of these diseases. Some biomarkers have been identified over the years and used in order to monitor disease activity, evaluate the efficacy of treatments, and prevent complications [5]. In a study [27] conducted on 105 patients with CD ad 98 patients with UC who collected blood samples for sCP levels and routine laboratory tests, the authors correlated values with C-reactive protein (CRP) and found that levels of sCP were higher during the active phase of CD and UC compared to controls but resulted in being more effective for evaluating patients affected by CD than UC. The diagnosis of IBD was based on a combination of clinical, radiological, endoscopic, and histological characteristics. The authors found that in patients with UC, sCP levels were comparable between active and inactive disease (regardless of the type of disease: left colitis, proctitis, or pancolitis). In patients with active CD, sCP levels were higher (regardless of the side of disease: ileocolitis, ileitis, or colitis) [27]. The results of the study demonstrated that sCP levels were higher in patients with CD and UC compared to normal controls. Moreover, in patients with CD, sCP levels were positively associated with laboratory parameters and clinical disease activity [27]. Another study of 171 patients (96 with IBD and 75 without IBD) reported that sCP correlated strongly with C-RP and fCP [26]. In a subgroup analysis of 50 patients with IBD, fCP was more effective in the diagnostic phase compared to sCP, but at 12-month follow-up, sCP was better at predicting treatment escalation and/or surgery for IBD patients (CD > UC). Therefore, the authors concluded that the combination of sCP and other blood biomarkers in a diagnostic and prognostic model could be predictive of IBD and its outcomes, including treatment escalation and surgery. Okata et al. [28], in a study of 101 IBD patients, found that the blood concentration of sCP was higher compared to healthy controls. The results showed that sCP was superior to levels of C-RP in revealing the severity of UC. Another study [29] evaluated a specific epitope (named CPa9-HNE) of sCP in patients with IBD to quantify neutrophil activity, showing accuracy in diagnosing IBD with severe activity. Serum CPa9-HNE levels were found to be four-fold higher in patients with UC and CD compared to healthy controls; these levels correlated with both the Simple Endoscopic Score and the Mayo score, revealing a strong association with disease activity [29]. Meuwis et al. [30] demonstrated that sCP was significantly higher in patients with active IBD compared with patients in remission (total patients: 115 affected by CD), concluding that sCP might be a useful biomarker for predicting relapse and/or response to therapy in patients with IBD (treated with infliximab). The authors reported significantly higher values of sCP in active gut disease compared to healthy controls (sCP of 8892 ng/mL in CD-patients vs. 1318 ng/mL in healthy controls) [30]. Ferrer et al. [4] performed a prospective study of 27 patients with UC and 26 patients with CD, concluding that sCP was a good indirect marker of gut inflammatory activity and there was a correlation with endoscopic findings in patients with UC; on the contrary, no statistically significant differences were found in patients with CD [4]. Reports on the pediatric population [31] assessed the role of sCP as a biomarker of IBD, underling a significant correlation with the disease activity scores, too. Carlesen et al. [32] conducted a pilot study in adolescents with IBD. The authors analyzed 19 UC samples and 49 CD samples, finding a correlation between sCP and endoscopic inflammation in patients with UC. The association between sCP, fCP, and symptom score was not significant. On the contrary, the association with CRP was significant. The authors concluded that sCP could have the potential to improve the monitoring of adolescents with UC. These findings confirmed that sCP is derived from circulating leukocytes, and in patients with autoimmune diseases, circulating neutrophils expressed more cell-surface calprotectin with a subsequent increase in its blood concentration. The studies are summarized in Table 1. 

### 3.3. sCP vs. fCP in IBD

The studies in the literature reported controversial results as regards the correlation between sCP and fCP. A study by Kalla et al. [26] underlines that sCP strongly matched fCP [33] and was a good predictor of IBD. In a subgroup of 50 patients, sCP was better than fCP in discriminating IBD from controls. At 11-month follow-up, sCP was better at predicting treatment escalation and/or surgery in patients with IBD, in particular CD. The authors concluded that sCP accurately predicted the inflammatory burden in patients affected by IBD. Further, it was able to predict disease burden and its outcomes [26]. On the other hand, some authors suggested a more significant correlation between serum calprotectin and serum C-RP but not fCP, suggesting that sCP was a more representative marker of systemic inflammation than localized gut inflammation if compared to fCP [8,27]. 

### 3.4. Methods and Costs of sCP and fCP Analysis

sCP and fCP can be evaluated using the enzyme-linked immunosorbent assay (ELISA) and a point-of-care test (POCT) [34,35]. ELISA methods for calprotectin analysis have been widely validated even if the protocol is quite time-consuming and the results are reported later than in POCT methods. To evaluate sCP patients, a blood sample (0.5 mL) must be collected in tubes containing ethylene-diamino-tetra-acetic acid. The sample is centrifuged for about 10 min at 10,000 rpm and then the extracted serum is collected and frozen at −20 °C. An amount of 100 µL of serum is diluted 1:50 and incubated at room temperature for 45 min. The plate is washed three times with diluted solution and then 100 µL of monoclonal anti-calprotectin antibodies conjugated with peroxidase is added. Then, a second washing procedure has to be performed, and the sCP concentration is calculated in µg/mL. As regards the analysis with POCT, immunochromatographic technology is inserted in a lateral flow assay system where a primary monoclonal antibody that targets calprotectin is used. The dilution is 1:1600 with an incubation time of 50 min. Then, a second antibody is used at a dilution of 1:600 (but with an incubation time of 15 min). These sections are developed using a 3,3′-diaminobenzidine for anti-calprotectin. The analysis of fCP can be realized by collecting stool samples and using an immunochromatographic assay for the rapid quantitative determination of calprotectin in patients’ stool. There are different immunochromatographic assays. They are non-invasive tests that allow the determination of, in less than thirty minutes, the level of fCP with quantitative and accurate analysis. One of these immunochromatographic assays, for example, uses a solid support (card) on which the patient’s stool sample has to be placed. Then, colored bands appear in the card; they are detected and quantified by a special reader. The quantification of fCP is performed by referring to a standard curve residing inside the reader. A normal value is considered less than 50 mg/Kg. Samples with an fCP concentration of more than 50 mg/kg are considered positive for the test. A positive test correlates with gut inflammation. fCP levels measured using an ELISA test resulted to be well correlated with fCP measured using the POCT, but they were not correlated with sCP levels. Patients with both UC and CD had higher neutrophil and macrophage expression of calprotectin compared with healthy subjects. fCP is considered a reliable marker for IBD’s disease activity, with a more rapid and simple measurement via POCT in clinical settings [36,37,38].

**Table 1:** sCP as biomarker of inflammatory bowel disease activity.

Authors	Study	Aim	Results
**Lugering et al. (1995)** [36]	62 CD	Authors evaluated the role of sCP in disease relapse	sCP levels were significantly increased in patients with active disease
**Leach et al. (2007)** [6]	29 CD, 4 UC	sCP in the assessment of disease burden	sCP indicated disease activity and elevated levels may contribute to the pathogenesis of IBD
**Meuwis (2013)** [30]	115 CD	The aim was to evaluate the value of sCP as a biomarker for CD	As a CD biomarker, sCP is complementary to fCP for prediction of relapse after infliximab withdrawal
**Hare et al. (2013)** [37]	45 UC	Role of sCP in the evaluation of prognosis	In the setting of acute severe UC, sCP is comparable with serum CRP in predicting outcomes
**Kalla et al. (2016)** [26]	35 CD, 45 UC	Role of sCP in diagnosis and prognosis	sCP combined with other blood-based biomarkers predicted disease and its outcomes
**Fukunaga et al. (2018)** [38]	13 CD, 41 UC	Authors quantified calprotectin levels in patients with IBD	Patients with CD and UC had higher neutrophil and macrophage calprotectin-positive expression levels
**Suarez Ferrer (2019)** [4]	27 UC, 26 CD.	A simple, reliable, and non-invasive biomarker is needed to enable the early detection of IBD inflammatory activity for correct management	sCP is a good indirect marker of IBD inflammatory activity and there was a correlation with endoscopic findings in UC. No significant differences in the case of CD
**Carlesen (2019)** [32]	20 UC	The measurement of fCP is limited by day-to-day variation and by feces consistency. Young patients are often noncompliant in collecting fecal samples, leading to suboptimal monitoring	sCP correlates with endoscopically assessed inflammation in UC
**Malham (2019)** [7]	54 IBD	To evaluate the role of sCP as a biomarker in IBD	sCP can be used as a biomarker of IBD disease activity, especially in cases where patients are not compliant in collecting fCP

## 4. Discussion

IBDs are chronic inflammatory disorders with an uncertain etiology. Genetic and environmental factors are involved in the etiology of IBD by interacting with the intestinal mucosa, gut bacteria, and immune cells [1,2,3,4]. Both UC and CD have remitting and relapsing phases. The initial onset of IBD is unknown, and the factors leading to relapse are also uncertain [1,2,3,4,5]. Some biomarkers have been identified over the years and used in order to diagnose and monitor disease activity, to evaluate the efficacy of treatments, and to prevent complications [26,27,28]. To date, interest in blood-based biomarkers is growing, since they are more suitable for patients and more convenient in their daily routine, compared to the collection of other biological samples (i.e., fecal samples). Few publications are available about the role of sCP as a circulating biomarker in IBD patients, different from other autoimmune diseases [5,26,27,28,29,30,31,32]. Recent investigations have shown that sCP can be a valuable biomarker for the diagnosis, prognosis, and evaluation of IBD’s disease burden. Even if the majority of serum biomarkers (IL-6, IL-8, TNF-α…) currently available have limited specificity for IBD, the dosage of sCP has been proposed as an effective aid both in the management and in predicting long-term outcomes in patients affected by UC and CD [5,26,27,30]. The data in the literature from several studies show that sCP resulted in an increase in active IBD and it was correlated with disease activity in both UC and CD. Furthermore, a significant correlation has been identified with CRP levels and with endoscopic findings and scores, such the simple endoscopic score for Crohn’s disease [SES-CD] and the Mayo score [8,25,26,30,31]. Moreover, a positive correlation has also been found with clinical indices such as the Harvey and Walmsley index [4]. sCP originates from leukocytes in response to many stimuli both in the bloodstream and in the gut. Neutrophils are usually considered as first responders in inflammation, including bowel inflammation. Research studies demonstrate that sCP is released by circulating neutrophils and monocytes and by macrophages in response to lipopolysaccharides (LPSs), TNF-alpha, and interleukin1β, being a key marker of systemic inflammation [27]. Cytokines play a pivotal role in the immunopathogenesis of UC and CD, and they can stimulate the production of sCP [4,5,26,32]. sCP has been positively correlated with CRP as regards the detection of intestinal inflammation, and it could be helpful in differentiating between different endoscopic diseases’ activity in patients with IBD in a minimally invasive manner [27]. In some patients with active IBD, it has been shown to have a higher sensitivity compared with CRP, providing more relevant information about the extension of the inflammatory response [27]. The role of sCP has been better characterized in autoimmune rheumatological diseases, with a pivotal role as an activator of innate immunity; in fact, it triggers chemotaxis, the migration of neutrophils, and the modulation of macrophages, leading to inflammation [16,17,18,39]. In this context, it is known that some immune-mediated diseases can coexist and extend from one site to another. In fact, it is estimated that about 20% of patients affected by IBD develop presentations including immune-mediated forms of arthritis [13,20], uveitis, and other gastrointestinal and dermatological diseases [40,41,42]. So, the analysis of sCP could be useful in identifying the degree of activity in immune-mediated diseases (from sacro-iliac joint inflammation and other forms of arthritis to gut inflammation). Over the years, the majority of studies have been conducted on fCP to score IBD’s disease activity [34]. Studies have shown contrasting results in the correlation with CRP, considering fCP more sensitive and specific for the diagnosis of IBD, its disease activity, and relapse prediction after treatment. Few studies focus on the association between fCP and sCP in patients with UC and CD. fCP is considered a more direct biomarker for intestinal inflammation and mucosal healing [17,27,43,44], while sCP plays a pivotal role in systemic inflammation. Based on the fact that the incidence of IBD is increasing, the use of blood tests could be simpler for patients and give physicians an easier tool for diagnosis and follow-up. The analysis of stool samples, in some situations, can be challenging due to both laboratory workload and preanalytical variations, and young patients are not always compliant in collecting stool samples. The easy measure of sCP can be a promising in detecting gut inflammation and selecting patients for endoscopy. In addition, it could be useful to monitor patients in both short and long periods of time and schedule the timing of endoscopic controls [26,28]. Veyrard et al. [45] used sCP to follow 119 IBD patients at three and six months in the period of disease relapse who were being treated with biologic therapies. The median sCP levels at baseline were 3.15 µg/mL; in cases of relapse, this value grew to 4.45 µg/mL. The authors concluded that sCP was significantly higher in patients with clinical relapse compared to those with endoscopic remission (regardless of clinical symptoms). sCP allowed patients with active IBD to be identified but failed to predict relapse. A weak correlation was reported between the levels of sCP and fCP. To date, more studies are needed to promote the routine use of sCP in clinical practice; identify a standardized cut-off for diagnosis, relapse, and prognosis; and use it as a diagnostic and prognostic biomarker as a replacement for the most commonly studied fCP. 

## 5. Conclusions

sCP could represent a useful complementary biomarker in the evaluation of the inflammatory stage of patients with immune-mediated gastrointestinal and non-gastrointestinal diseases. In particular, in our opinion, sCP can be used as a better marker of systemic inflammation than localized gut inflammation. It can also be helpful in non-gastrointestinal diseases, such as for the evaluation of arthritis involvement in IBD patients and joint inflammation in patients with autoimmune diseases such as RA, SLE, and axSpA. More studies are needed to promote its routine use in clinical practice as a diagnostic and prognostic biomarker for gastrointestinal diseases, together with fCP, according to the different kinds of patients.

## Data Availability

No new datasets were created or analyzed in this study. Data sharing is not applicable to this article.

## References

[1] Moore B.W. (1965). A soluble protein characteristic of the nervous system. Biochem. Biophys. Res. Commun..

[2] Jukic A., Bakiri L., Wagner E.F., Tilg H., Adolph T.E. (2021). Calprotectin: From biomarker to biological function. Gut.

[3] Pathirana W.G.W., Chubb S.P., Gillett M.J., Vasikaran S.D. (2018). Faecal Calprotectin. Clin. Biochem. Rev..

[4] Suárez-Ferrer C., Abadía B.M., García R.L., Poza C.J., Jaquotot H.M., Cerpa A.A., Martín A. (2019). The use of serum calprotectin as a biomarker for inflammatory activity in inflammatory bowel disease. Rev. Esp. Enferm. Dig..

[5] Kopi T.A., Shabnam S., Shahrokh M., Hamid A.A., Azade A.K. (2019). The role of serum calprotectin as a novel biomarker in inflammatory bowel diseases: A review study. Gastroenterol. Hepatol. Bed Bench.

[6] Leach S.T., Yang Z., Messina I., Geczy C.L., Cunningham A.M., Day A.S. (2007). Serum and mucosal S100 proteins, calprotectin (S100A8/S100A9) and S100A12, are elevated at diagnosis in children with inflammatory bowel disease. Scand. J. Gastroenterol..

[7] Malham M., Carlsen K., Riis L., Paerregaard A., Vind I., Fenger M., Wewer V. (2019). Plasma calprotectin is superior to serum calprotectin as a biomarker of intestinal inflammation in ulcerative Colitis. Scand. J. Gastroenterol..

[8] Carnazzo V., Redi S., Basile V., Natali P., Gulli F., Equitani F., Marino M., Basile U. (2024). Calprotectin: Two sides of the same coin. Rheumatology.

[9] Garcia V., Perera Y.R., Chazin W.J. (2022). A Structural Perspective on Calprotectin as a Ligand of Receptors Mediating Inflammation and Potential Drug Target. Biomolecules.

[10] Nanini H.F., Bernardazzi C., Castro F., de Souza H.S.P. (2018). Damage-associated molecular patterns in inflammatory bowel disease: From biomarkers to therapeutic targets. World J. Gastroenterol..

[11] Donato R. (2003). Intracellular and extracellular roles of S100 proteins. Microsc. Res. Tech..

[12] Atreya R., Neurath M.F. (2015). Molecular pathways controlling barrier function in IBD. Nat. Rev. Gastroenterol. Hepatol..

[13] Kopeć-Mȩdrek M., Widuchowska M., Kucharz E.J. (2016). Calprotectin in rheumatic diseases: A review. Reumatologia.

[14] Chatzikonstantinou M., Konstantopoulos P., Stergiopoulos S., Kontzoglou K., Verikokos C., Perrea D. (2016). Calprotectin as a diagnostic tool for inflammatory bowel diseases. Biomed. Rep..

[15] Manfredi M., Van Hoovels L., Benucci M., De Luca R., Coccia C., Bernardini P., Russo E., Amedei A., Guiducci S., Grossi V. (2023). Circulating Calprotectin (cCLP) in autoimmune diseases. Autoimmun. Rev..

[16] Inciarte-Mundo J., Frade-Sosa B., Sanmartí R. (2022). From bench to bedside: Calprotectin (S100A8/S100A9) as a biomarker in rheumatoid arthritis. Front. Immunol..

[17] Ma Y., Fan D., Xu S., Deng J., Gao X., Guan S., Pan F. (2020). Calprotectin in spondyloarthritis: A systematic review and meta-analysis. Int. Immunopharmacol..

[18] Sejersen K., Weitoft T., Knight A., Lysholm J., Larsson A., Rönnelid J. Serum calprotectin correlates more strongly with inflammation and disease activity in ACPA positive than ACPA negative rheumatoid arthritis. Rheumatology.

[19] Saut A., Paclet M.H., Trocmé C., Toussaint B., Bocquet A., Bouillet L., Baillet A. (2023). Serum calprotectin is a marker of disease activity in Giant cell arteritis. Autoimmun. Rev..

[20] Klingberg E., Carlsten H., Hilme E., Hedberg M., Forsblad-d’Elia H. (2012). Calprotectin in ankylosing spondylitis–frequently elevated in feces, but normal in serum. Scand. J. Gastroenterol..

[21] Ercalik C., Baskaya M.C., Ozdem S., Butun B. (2021). Investigation of asymptomatic intestinal inflammation in ankylosing spondylitis by fecal calprotectin. Arab. J. Gastroenterol..

[22] Radwan A.R., Allam A., Radwan A. (2021). The relationship of serum calprotectin with disease activity, functional status, ultrasonographic findings and radiological damage in rheumatoid arthritis patients. Egypt. Rheumatol..

[23] Gernert M., Schmalzing M., Hans-Peter T., Strunz P.P., Schwaneck E.C., Fröhlich M. (2022). Calprotectin (S100A8/S100A9) detects inflammatory activity in rheumatoid arthritis patients receiving tocilizumab therapy. Arthritis Res. Ther..

[24] Haga H.J., Brun J.C., Graham R.V. (1993). Calprotectin in Patients with Systemic Lupus Erythematosus: Relation to Clinical and Laboratory Parameters of Disease Activity. Lupus.

[25] Poullis A., Foster R., Mendall M.A., Fagerhol M.K. (2003). Emerging role of calprotectin in gastroenterology. J. Gastroenterol. Hepatol..

[26] Kalla R., Kennedy N.A., Ventham N.T., Boyapati R.K., Adams A.T., Nimmo E.R., Visconti M.R., Drummond H., Ho G.T., Pattenden R.J. (2016). Serum Calprotectin: A Novel Diagnostic and Prognostic Marker in Inflammatory Bowel Diseases. Am. J. Gastroenterol..

[27] Mori A., Mitsuyama K., Sakemi R., Yoshioka S., Fukunaga S., Kuwaki K., Yamauchi R., Araki T., Yoshimura T., Yamasaki H. (2021). Evaluation of Serum Calprotectin Levels in Patients with Inflammatory Bowel Disease. Kurume Med. J..

[28] Okada K., Okabe M., Kimura Y., Itoh H., Ikemoto M. (2019). Serum S100A8/A9 as a Potentially Sensitive Biomarker for Inflammatory Bowel Disease. Lab. Med..

[29] Mortensen J.H., Sinkeviciute D., Manon-Jensen T., Domislović V., McCall K., Thudium C.S., Brinar M., Önnerfjord P., Goodyear C.S., Krznarić Ž. (2022). A Specific Calprotectin Neo-epitope [CPa9-HNE] in Serum from Inflammatory Bowel Disease Patients Is Associated with Neutrophil Activity and Endoscopic Severity. J. Crohns Colitis.

[30] Meuwis M.A., Vernier-Massouille G., Grimaud J.C., Bouhnik Y., Laharie D., Piver E., Seidel L., Colombel J.F., Louis E. (2013). Serum calprotectin as a biomarker for Crohn’s disease. J. Crohns Colitis.

[31] Herrera O.R., Christensen M.L., Helms R.A. (2016). Calprotectin: Clinical Applications in Pediatrics. J. Pediatr. Pharmacol. Ther..

[32] Carlsen K., Malham M., Hansen L.F., Petersen J.J.H., Paerregaard A., Houen G., Wewer V. (2019). Serum Calprotectin in Adolescents with Inflammatory Bowel Disease-A Pilot Investigation. J. Pediatr. Gastroenterol. Nutr..

[33] Hovstadius H., Lundgren D., Karling P. (2021). Elevated Faecal Calprotectin in Patients with a Normal Colonoscopy: Does It Matter in Clinical Practice? A Retrospective Observational Study. Inflamm. Intest. Dis..

[34] Khaki-Khatibi F., Qujeq D., Kashifard M., Moein S., Maniati M., Vaghari-Tabari M. (2020). Calprotectin in inflammatory bowel disease. Clin. Chim. Acta.

[35] Ojetti V., Saviano A., Covino M., Acampora N., Troiani E., Franceschi F., GEMELLI AGAINST COVID-19 group (2020). COVID-19 and intestinal inflammation: Role of fecal calprotectin. Dig. Liver Dis..

[36] Lügering N., Stoll R., Kucharzik T., Schmid K.W., Rohlmann G., Burmeister G. (1995). Immunohistochemical distribution and serum levels of the Ca^2+^-binding proteins MRP8, MRP14 and their heterodimeric form MRP8/14 in Crohn’s disease. Digestion.

[37] Hare N., Kennedy N., Kingstone K., Arnott I., Shand A., Palmer K. (2013). PTH-082 Serum Calprotectin: A Novel Biomarker to Predict Outcome in Acute Severe Ulcerative Colitis?. Gut.

[38] Fukunaga S., Kuwaki K., Mitsuyama K., Takedatsu H., Yoshioka S., Yamasaki H. (2018). Detection of calprotectin in inflammatory bowel disease: Fecal and serum levels and immunohistochemical localization. Inter. J. Mol. Med..

[39] Shi-Jun H., Zhang-Nan D., Hui-Xian X., Lin F. (2020). Association Between Serum S100A8/S100A9 Heterodimer and Pulmonary Function in Patients with Acute Exacerbation of Chronic Obstructive Pulmonary Disease. Lung.

[40] Lee Y.G., Jisu H., Lee P.H., Junehyuk L., DoJin K., An-Soo J. (2020). Serum Calprotectin Is a Potential Marker in Patients with Asthma. J. Korean Med. Sci..

[41] Dumitrascu D.L. (2022). Serum calprotectin: A new potential biomarker for psoriasis?. Minerva Med..

[42] Kang K.Y., Park S.H., Hong Y.S. (2020). Relationship between faecal calprotectin and inflammation in peripheral joints and entheses in axial spondylarthritis. Scand. J. Rheumatol..

[43] Yasuda R., Arai K., Kudo T., Nambu R., Aomatsu T., Abe N., Kakiuchi T., Hashimoto K., Takahashi M., Yamashita Y. (2023). Serum leucine-rich alpha-2 glycoprotein and calprotectin in children with inflammatory bowel disease: A multicenter study in Japan. J. Gastroenterol. Hepatol..

[44] Sands B.E. (2015). Biomarkers of Inflammation in Inflammatory Bowel Disease. Gastroenterology.

[45] Veyrard P., Roblin X., Pansart C., Mao R., Nancey S., Killian M., Williet N., Bastide L., Tournier Q., Paul S. (2022). Serum calprotectin is useful to confirm inflammatory bowel disease activity but not to predict relapse. Clin. Immunol..

